# *nifH* gene expression and diversity in geothermal springs of Tengchong, China

**DOI:** 10.3389/fmicb.2022.980924

**Published:** 2022-09-08

**Authors:** Zhao-Qi Song, Li Wang, Feng Liang, Qingfeng Zhou, Dongli Pei, Hongchen Jiang, Wen-Jun Li

**Affiliations:** ^1^College of Biology and Food, Shangqiu Normal University, Shangqiu, China; ^2^State Key Laboratory of Biogeology and Environmental Geology, China University of Geosciences, Wuhan, China; ^3^State Key Laboratory of Biocontrol, Guangdong Key Laboratory of Plant Resources, School of Life Sciences, Sun Yat-sen University, Guangzhou, China

**Keywords:** diazotrophs, *nifH* gene, *in situ* expression, hot spring, Tengchong

## Abstract

Terrestrial hot springs have been suggested to harbor diverse diazotrophic lineages by using DNA-based *nifH* gene phylogenetic analysis. However, only a small amount of diazotrophs were ever confirmed to perform nitrogen fixation. In order to explore the compositions of active diazotrophic populations in hot springs, the *in situ* expression and diversity of *nifH* and 16S rRNA genes were investigated in the sediments of hot springs (pH 4.3-9.1; temperature 34-84°C) in Tengchong, China, by using high-throughput sequencing. The results showed that active diazotrophs were diverse in the studied Tengchong hot springs. The main active diazotrophs in high-temperature hot springs were affiliated with *Aquificae*, while those in low-temperature hot springs belonged to *Cyanobacteria* and *Nitrospirae*. Such dominance of *Aquificae* and *Nitrospirae* of diazotrophs has not been reported in other ecosystems. This suggests that hot springs may harbor unique active diazotrophs in comparison with other type of ecosystems. Furthermore, there were significant differences in the phylogenetic lineages of diazotrophs between hot springs of Tengchong and other regions, indicating that diazotrophs have geographical distribution patterns. Statistical analysis suggests that the expression and distribution of *nifH* gene were influenced by temperature and concentrations of ammonia and sulfur seem in Tengchong hot springs. These findings avail us to understand element cycling mediated by diazotrophs in hot spring ecosystems.

## Introduction

Biological dinitrogen (N_2_) fixation, as an important nitrogen source of nitrogen-limited ecosystems, provides bioavailable fixed nitrogen for the synthesis of biological macromolecules, metabolism, and cell growth of microbial community (Falkowski, 1997; Zehr et al., 2003). Nitrogen is usually limited in terrestrial geothermal springs due to the rapid assimilation and transformation of inorganic nitrogen (Estrella-Alcamán et al., 2015; Lin et al., 2015). Therefore, nitrogen fixation processes may exist to support microbial demand for nitrogen in hot springs.

With the use of DNA-based methods, *nifH* gene coding for the ferric protein subunit of the nitrogenase complex of diazotrophs has been proved to be widespread in hot springs (temperature: 35-84°C; pH: 2-9) of Yellowstone National Park (YNP, United States) (Miller et al., 2006; Hall et al., 2008; Hamilton et al., 2011a). Subsequently, *nifH* gene was also detected in hot springs in Porcelana (Chile; Estrella-Alcamán et al., 2015), Nakabusa (Japan; Nishihara et al., 2018a) and Iceland (Cousins et al., 2018). These previous studies showed that hot spring diazotrophs are diverse and mainly belong to *Cyanobacteria*, *Proteobacteria*, *Firmicutes*, *Chloroflexi*, *Aquificae*, and *Nitrospirae*. However, the DNA-based methods can only indicate the presence of diazotrophs, but cannot reveal their activity and whether they promote nitrogen fixation process in hot springs. To confirm the presence of active diazotrophs, *in situ* expression of *nifH* gene and(or) nitrogenase has been investigated in some YNP hot springs (Steunou et al., 2006, 2008; Hamilton et al., 2011b,^2014^; Loiacono et al., 2012) and Porcelana (Estrella-Alcamán et al., 2015; Alcamán-Arias et al., 2018). Generally, two cyanobacterial genera, i.e., *Mastigocladus* and *Synechococcus*, were confirmed as active diazotrophic taxa in hot springs with photosynthesis (Steunou et al., 2006, 2008). In contrast, in chemotrophic hot springs, most *nifH* gene transcripts were related to the *Aquificae* genera of *Thermocrinis* and *Hydrogenobacter*, while only a few *nifH* gene transcripts were affiliated with *Nitrospirae* (genus *Thermodesulfovibrio*) and *Chloroflexi* (genus *Roseiflexus*). These previous results indicated that active diazotrophs of hot springs were just limited to a few taxa. However, considering the high diversity of diazotrophs revealed by DNA-based studies, it is reasonable to hypothesize that more undetected groups of diazotrophs may play an important role in nitrogen fixation process in hot springs.

In addition, one interesting finding of previous *in situ* studies is that *Aquificae*-related *nifH* gene transcripts dominate in the YNP high-temperature hot springs, but have never been reported to appear in other types of environments (Loiacono et al., 2012; Hamilton et al., 2014). This suggests that the composition of diazotrophs is very unique in terrestrial hot springs. Indeed, *Aquificae*-related bacteria widely exists in terrestrial hot springs with high temperature chemosynthetic function and play important roles in the cycling of carbon, sulfur and hydrogen (Hedlund et al., 2015). These above findings suggest *Aquificae* may also be the main executors of nitrogen fixation in global hot springs with chemosynthesis. This speculation was supported by the retrieval of two *Aquificae* strains capable of nitrogen fixation isolated from one hot spring in Nakabusa, Japan (Nishihara et al., 2018b). But more evidence is needed to prove the expression of *nifH* gene by *Aquificae*.

Temperature is regarded as one important factor shaping the microbial community of hot springs (Meyer-Dombard et al., 2005; Hou et al., 2013). In normal environments, multiple factors are related to the distribution of diazotrophs. For example, pH and available carbon and nitrogen shape the diazotrophic composition in agricultural soils of China (Hu et al., 2021) and Argentina (Calderoli et al., 2017), while oxygen and iron affect the active diazotrophic lineages in some types of aquatic habitats (Moisander et al., 2014; Larson et al., 2018). However, the effect of temperature and other environmental factors on active diazotrophs in hot spring remains unclear.

There are thousands of hot springs with various hydrothermal characteristics in the Tengchong geothermal zone in Yunnan Province of southwestern China, one of the most active geothermal areas in the world (Du et al., 2005). Previous 16S rRNA gene-based studies showed that Tengchong hot springs were inhabited by highly diverse prokaryotic communities and harbored many potential diazotrophic groups (e.g., *Synechococcus*, *Hydrogenobacte*r). Many of these groups are unique lineages that have never been detected in hot springs of other regions (Song et al., 2012; Jiao et al., 2020, 2021; Xian et al., 2020). One DNA-based study revealed that the *nifH* gene composition in two Thengchong hot springs has some common and unique phylogenetic lineages (Feng et al., 2018). However, there is still a lack of comprehensive census on the difference of active diazotrophs among different geothermal regions. Such knowledge gap limits our understanding of the distribution and adaptability of diazotrophs in global hot springs.

In this study, the expression and diversity of *nifH* and 16S rRNA genes were investigated in geothermal springs with a wide range of temperature and pH in Tengchong. An integrated approach including high-throughput sequencing and geochemical analyses were employed. The main aims of this study are to investigate (1) the composition and distribution of active diazotrophs in Tengchong geothermal springs; (2) the differences of diazotrophic lineages among different geothermal regions; and (3) the influences of environmental variables on the active diazotrophic communities.

## Materials and methods

### Field measurements and sampling

Sampling cruise was performed in Aug 2014. A total of 20 hot springs in Tengchong were selected for field measurements and sampling (Supplementary Table 1 and Supplementary Figure 1). At each hot spring, field measurement and sampling were performed during 9 am-13 pm every day. In the field, water temperature and pH were determined using a Hach pH meter equipped with a pH probe and a temperature probe (PT-10, SARTORIUS, Germany). Nitrite, nitrate, ammonium and sulfide were measured using Hach kits (model CEL 850/product #: 2687900, Hach Chemical Co., Iowa, United States) according to the manufacturer’s instructions. After field measurement, streamers, mat-containing sinters and sediments in different sites of each sampled hot spring were collected and mixed, then were put into 2 ml centrifuge tubes and 15 ml Falcon tubes, respectively. The sample tubes were immediately stored in liquid nitrogen. The samples were kept in liquid nitrogen in the field, during transportation and stored in the laboratory until further RNA extraction.

### RNA extraction and cDNA synthesis

Environmental RNA was extracted from 1 to 3 gram of sediment, mat or sinter samples using the PowerSoil RNA Isolation Kit (Mo BIO Laboratories, Carlsbad, CA, United States). To remove the residual DNA, the soluble crude RNA was digested with RNase-free DNase I (Fermentas, United States). The DNase-digested RNA samples were checked for potential genomic DNA contamination by PCR amplification with specific primer sets of 16S rRNA and *nifH* genes (see below for primer information).

The checked RNA samples were quantified using Nanodrop UV-Vis spectrophotometer (ThermoFisher, United States) and reverse transcribed into cDNA using the Fermentas AMV Reverse Transcriptase (Fermentas, United States). Absence of potential contamination from DNA and chemical reagents was verified by conducting the same reactions without the AMV reverse transcriptase and template, respectively.

### Amplification and sequencing of 16S rRNA and *nifH* genes

The resulting cDNA was employed as the template for amplification of polymerase chain reactions (PCR) of 16S rRNA and *nifH* genes. The hypervariable V4 region of the 16S rRNA genes were PCR amplified using primer sets of 515F (5′-GTG CCA GCM GCC GCG GTA A-3′) and 806R (5′-GGA CTA CHV GGG TWT CTA AT-3′) (Caporaso et al., 2011). The fragments of *nifH* genes were PCR amplified using specific primer sets of KAD3 (5′-ATH GTI GGI TGY GAY CCI AAR GCI GA-3′) and DVV (5′-ATI GCR AAI CCI CCR CAI ACI ACR TC-3′) (Gaby and Buckley, 2012). To pool multiple samples for one run of high-throughput sequencing, a sample tagging approach was used (Meyer et al., 2008). Each tag was added to the 5′ end of the reveres primer of 16S rRNA gene and the forward primer KAD3 of *nifH* gene, respectively. PCR amplification of 16S rRNA gene consisted of an initial denaturation at 95°C for 5 min, 25 cycles of denaturation at 95°C for 45 s, annealing at 54°C for 1 min, and extension at 72°C for 1 min, and a final extension at 72°C for 10 min. Individual reagents and their concentrations were as follows: 1 × PCR buffer with 1.5 mM Mg_2_Cl, dNTPs (100 M each), 0.25 M each primer, 2.5 U of DNA polymerase (Ex-Taq) (TaKaRa, Dalian, China) and 1ul of cDNA. The PCR amplification of *nifH* was 35 cycles and annealing temperature was 56°C, and the other protocols were same as the 16S rRNA gene. PCR products were purified using a QIAquick Gel Extraction Kit (QIAGEN, Germany). The purified PCR products were annealed to oligonucleotides that are complementary to an adaptor sequence. Sample libraries were generated from purified PCR products, and subsequently were pooled in equimolar concentrations and added library specific sequencing adapters by NEBNext Ultra (NEB#e7370S/L) assay as followed by instruction, and dual index sequencing of paired-end 250 bp was run on an Illumina Hiseq2500 instrument (Illu- mina, San Diego, CA, United States). The sequencing process was completed in Fixgene Theh Co., Ltd. (Beijing, China).

Forward and reverse reads were merged into full-length sequences using FLASH (Magoč and Salzberg, 2011). The raw sequence data were evaluated and filtered to ensure that >80% of the base calls in a sequence had a Phred quality score of 20 using the FASTQ Quality Filter (*q* = 20, *p* = 0.8). Then the barcode and primer sequences were deleted using FLEXBAR (Roehr et al., 2017). Sequences were removed if they were below certain length (16S rRNA gene < 200bp, *nifH* gene < 250bp) or contained ambiguous bases. Chimeric sequences were identified and discarded using the USEARCH software (Edgar et al., 2011).

### The analysis of 16S rRNA gene

A total of 17,000 sequences of 16S rRNA gene were extracted randomly from the high-quality sequence of each sample for the following analysis. The operational taxonomic units (OTUs) of 16S rRNA gene were classified using UCLUST at the 97% similarity level. OTUs that contain less than two sequences were removed. Sequences were taxonomically classified by the Silva database (db128) using RDP algorithm (60% threshold) (Quast et al., 2013). Potential contamination sequences (e.g., unknown, chloroplast, mitochondria, etc.) were removed. The alpha diversity estimates, including coverage (Jiang et al., 2006), Shannon-Wiener’s diversity index (Spellerberg and Fedor, 2003) and Simpson’s diversity index (He and Hu, 2005) were calculated by using Mothur software (Schloss et al., 2009). The *nifH* gene sequences (a total of 6,243 sequences) that can be identified as the main taxa at the genus level in GenBank were selected, with no terminator in the sequence and a length greater than 600bp as the standard. These selected sequences were employed to construct a local database including potential nitrogen fixation activities, based on the classification of these sequences. By Blasting against the constructed local database, the genus with potential nitrogen fixation activity in 16Sr RNA Gene Data in this study was determined.

The PICRUSt (Langille et al., 2013) and Piohillin (Iwai et al., 2016) (with 99% identity cut-off) were applied to predict the abundance of *nifH* gene-related 16S rRNA-derived OTUs. For the PICRUSt analysis, the OTU table was normalized by dividing each OTU abundance by the number of the known or predicted 16S rRNA gene copies. Subsequently, the weighted NSTI score was used to measure the phylogenetic distance between OTUs and reference OTUs in each sample, and to evaluate the prediction accuracy of PICRUSt. The final functions for metagenome were predicted with the script predict_ metagenomes.py, generating a table of Kyoto Encyclopedia of Genes and Genomes (KEGG) Orthologs (KOs). Thirdly, the predicted metagenomes were classified into higher categories (e.g., KOs into KEGG pathways). The Piohillin analysis was performed on the website^1^ according to the pipeline, and the reference database was used the “BioCyc”.

### The analysis of *nifH* gene

The *nifH* gene sequences were translated into amino acid sequences by using RDP Framebot^2^, and the sequences containing in-frame stop codon were excluded. Subsequently, the remaining sequences were used for OTU analysis with 95% similarity as the cutoff value by using USEARCH software. The alpha diversity estimates, including coverage, Shannon-Wiener’s diversity index, and Simpson’s index of diversity, were calculated by using Mothur software (Schloss et al., 2009). The beta diversity analysis was conducted using QIIME2 (Bolyen et al., 2019), with the script core_diversity_analyses.py. Jackknifed weighted pair group method with arithmetic mean (UPGMA) clustering was performed to compare microbial community similarity among the studied samples based on UniFrac weighted phylogenetic distances.

The obtained *nifH* gene sequences were classified into OTUs by the phylogenetic analysis combined with representative sequences of known diazotrophic lineages (refer to BLAST results in Non-Redundant Protein Sequence Database and the papers of Zehr et al., 2003 and Raymond et al., 2004). According to the published accession numbers, the *nifH* gene sequences isolated from hot springs of other regions were downloaded and clustered into OTUs at the 95% similarity level. The sequences of *nifH* gene OTUs from this study and other regions were combined for the phylogenetic analysis. The Neighbor-joining phylogenetic trees were constructed from dissimilar distance and pairwise comparisons with the Jukes-Cantor distance model using the MEGA7.0 (molecular evolutionary genetics analysis) program. Bootstrap replications of 1,000 were assessed. The phylogenetic tree visualization was created by using the iTOL web server (Letunic and Bork, 2019).

To identify the differences in environmental background of hot springs, principal component analysis (PCA) of physicochemical variables was performed by using R software. Spearman’s correlation and redundancy analyses (RDA) were also performed to explore relationships of microbial community patterns with physicochemical variables. The significance of the RDA models and the explanatory factors were tested by using 999 permutations. The ordination on the x- and y-axis and the length of the corresponding arrows indicate the relative importance.

### Nucleotide sequence accession numbers

All the *nifH* and 16S rRNA gene sequences obtained from this study have been deposited at the National Omics Data Encyclopedia (NODE)^3^ under the project OEP003492 with run accession number OER258273 to OER258312.

## Results

### Sample description

The studied hot springs exhibited a wide range of physical and chemical conditions (Table 1). The temperature of the studied hot springs ranged 34-84°C with pH ranging 4.3-9.1; The concentrations of nitrite, nitrate and ammonium were below 8 mg/L (Table 1). Principal component analysis (PCA) and the Kruskal-Wallis test (*p* < 0.001) showed that the physicochemical variables differed significantly among the sampled hot springs (Supplementary Figure 2).

**TABLE 1:** Physicochemical variables of the investigated hot springs.

Sample name	Sample code	Temp (°C)	pH	NO_2_^–^ (mg/L)	NO_3_^–^ (mg/L)	NH_3_^+^ (mg/L)	S^2–^ (mg/L)
Hashang#1	Hs1	84	8.2	0.005	0.010	0.100	16.000
Shuirebaozha#2	Srbz2	84	8.2	BD	BD	0.080	7.000
Shuirebaozha#3	Srbz3	84	9.1	BD	0.010	0.230	17.000
Hamazui	Hmz	74	7.8	0.003	0.010	0.120	14.000
Hashang#2	Hs2	73	8.2	0.001	0.010	0.370	4.700
Huanxiquan#2	Hxq2	72	6.7	0.017	BD	0.090	21.000
Rehaitiyanqu#5	Rhtyq5	70	4.3	0.004	BD	BD	20.900
Xianrendong	Xrd	68	7.5	0.019	BD	0.020	0.330
Hashang#3	Hs3	66	8.2	0.013	0.017	0.070	0.150
Rehaitiyanqu#2	Rhtyq2	62	5.6	0.004	BD	0.010	24.200
Shizitou#1	Szt1	62	8.4	0.024	BD	5.460	0.490
Jinzequan	Jzq	61	7.2	BD	0.003	0.230	BD
Shuirebaozha#1	Srbz1	61	7	BD	0.013	0.100	0.127
Heinitan#2	Hnt2	56	7	0.007	BD	0.230	0.110
Jiemingquan	Jmq	56	7.8	0.011	0.030	0.160	7.100
Heinitan#3	Hnt3	54	6.8	0.051	0.020	1.260	0.041
Heinitan#1	Hnt1	53	7	0.007	BD	4.030	0.003
Huanxiquan#1	Hxq1	53	7	0.012	BD	7.700	0.045
Hashang#5	Hs5	52	7.2	0.022	BD	0.910	0.081
Shuirebaozha#4	Srbz4	34	6.7	0.003	0.020	0.340	BD

BD: below the detection limit (0.001 mg/L).

### Potential diazotrophic taxa based on 16S rRNA gene transcripts

**FIGURE 1:**
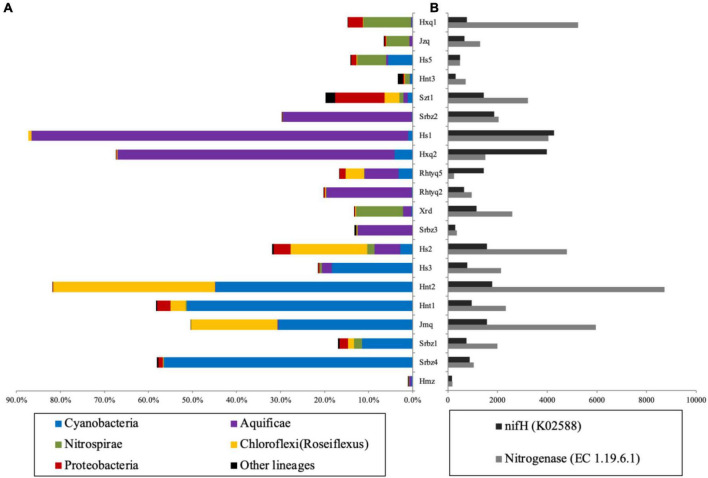
The predictive analysis of nitrogen fixing functions at the level of phylum, enzyme and gene based on the 16S rRNA gene data. Panel **(A)** showed the compositions of predicted potential diazotrophic taxa; Panel **(B)** showed the abundance of predicted nitrogenase and *nifH* gene. Note: Piphillin and PICRUSt were applied for the predictions of nitrogenase and *nifH* gene, respectively.

A total of 67 known prokaryotic phyla were identified, and only about 20 of them were dominant (their abundance accounted for above 5% of the total 16S rRNA gene sequences of each sample) in one hot spring. *Chloroflex*, *Proteobacteria*, *Aquificae*, *Cyanobacteria*, *Nitrospirae* and, *Acetothermia* were the most predominant phyla, and they dominated in more than six samples (Supplementary Figure 3). A total of 118 genus-level taxa with potential nitrogen fixation capacity were detected, and the abundance of 19 taxa was greater than 1% in more than one hot spring (Supplementary Figure 4). These identified taxa accounted for 80.7-99.9% of nitrogen-fixing organisms of the studied samples, and belonged to six phyla, i.e., *Cyanobacteria*, *Aquificae*, *Nitrospirae*, *Chloroflexi* and *Proteobacteria* (Figure 1A). *Aquificae* dominated in hot springs Srbz2, Hs1, Hxq2, Rhtyq5, Rhtyq2, Srbz3 (temperature: 62-84°C, pH: 4.3-9.1), accounting for 12-85% and 84-99% of the total 16S rRNA gene sequences and diazotrophic 16S rRNA gene sequences, respectively (Figure 1A). *Hydrogenobacter* is the only known genus with nitrogen fixation potential detected in *Aquificae* (Supplementary Figure 4). *Cyanobacteria* with photosynthetic function are dominant in Hs3, Hnt2, Hnt1, Jmq, Srbz, and Srbz4 hot springs (temperature: 34-66°C, pH: 7.0-8.2), accounting for 12-49% and 55-97% of total 16S rRNA gene sequences and diazotrophic 16S rRNA gene sequences, respectively (Figure 1A). The unclassified genus belonging to “Family I of subsection III” was one dominant cyanobacterial group, accounting for more than 10% of total 16S rRNA gene sequences in Hnt2, Jmq, and Srbz1 hot springs. Unclassified lineage belonging to “Family I of subsection I” was predominant cyanobacterial group in Srbz4 hot spring, accounting for 49% of the total 16S rRNA gene sequences (Supplementary Figure 4). *Thermosynechococcus* was the main genus of *Cyanobacteria* in Hs3, Hnt1, and Hnt2 hot springs, accounting for 18, 12, and 14.8% of total 16S rRNA gene sequences, respectively. *Nitrospirae* was the main potential diazotrophs (46 to 80% of the total diazotrophic 16S rRNA gene sequences) in Hxq1, Jzq, Hs5, Hnt3, and Xrd hot springs. The nitrogen fixation-associated *Nitrospirae* genus comprised of *Nitrospira* and *Thermodesulfovibrio*, with the former accounting for 46 and 71% of total 16S rRNA gene sequences in Hs5 and Hxq1 hot springs, respectively, and the latter accounting for 61, 77, and 80% of total 16S rRNA gene sequences in Hnt3, Jzq and Xrd hot springs, respectively (Supplementary Figure 4). *Proteobacteria* was detected in all the studied hot springs and covered 54 potential diazotrophic genera. However, only *Geobacter*, *Desulfonema* and *Desulfobulbus* belonging to *Deltaproteobacteria* and *Pseudoalteromonas* belonging to *Gammaporteobacteria* accounted for more than1% prokaryotic communities in more than one hot spring. The genera of *Geobacter* and *Pseudoalteromonas* accounted for 42% and 8% of diazotrophic community of Szt1 hot spring, respectively (Supplementary Figure 4).

### Prediction analysis of potential nitrogen fixation function based on 16S rRNA genes

The PICRUSt analysis showed the NSTI score of each sample ranged from 0.05 to 0.28 (Supplementary Table 2). This ensures the predicted results can provide some important insights into microbial functions according to previous studies (Langille et al., 2013; Wang et al., 2016; Fan et al., 2017). The abundance of predicted nitrogenase-related genes was higher than that of *nifH* gene in most of the studied hot springs, which may be due to different prediction methods. The relative abundance of *nifH* gene, nitrogenase, and the nitrogen-fixing taxa showed consistent variation trends (Figure 1B). The abundance of nitrogen-fixing taxa was correlated with the abundance of *nifH* gene (*r* = 0.69, *p* < 0.001) and nitrogenase (*r* = 0.50, *p* < 0.001), indicating that the analysis of diazotrophic community diversity predicted on the basis of 16S rRNA gene sequence was reliable.

### Phylogeny and composition of expressive *nifH* gene

**FIGURE 2:**
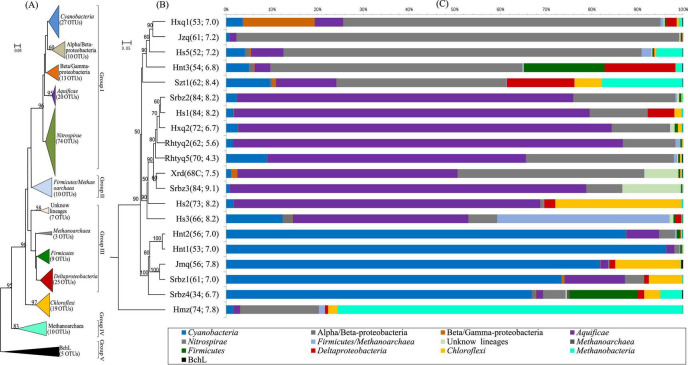
Phylogenetic analysis, UPGMA analysis and community composition of *nifH* gene in the investigated hot springs. **(A)** Neighbor-joining tree showing the phylogenetic affiliation of the OTUs based on protein sequences. Scale bars indicate the Jukes-Cantor distances. Bootstrap values of 50% (for 1,000 iterations) are shown; **(B)** UPGMA cluster tree based on Bray-Curtis dissimilarity. **(C)** Compositions of *nifH* gene. The numbers in the parentheses are the temperature and pH of each site, respectively.

A total of 1,384,410 high-quality *nifH* gene sequences were retrieved from the cDNA samples, and they can be classified into 1,587 OTUs at the 95% protein sequence similarity level (Bae et al., 2018). The *nifH* gene amplification with the DNase-treated RNA yielded negative results, which proved that the obtained *nifH* gene applications were derived from cDNA transcripts rather than DNA contamination. However, due to the highly sensitivity of high-throughput sequencing approaches (Sogin et al., 2006), the possibility of DNA source cannot be absolutely excluded for some OTUs with very low abundance. Thus, 230 OTUs (accounting for 95.3- 99.8% of the total *nifH* gene sequences in each hot spring) whose abundance higher than 0.1% in at least one hot spring were remained for the downstream phylogenetic analysis. The obtained *nifH* gene OTUs were divided into five major groups (I-V) (the phylogenetic types referred to Zehr et al., 2003 and Raymond et al., 2004) that comprised of thirteen lineages. The OTUs belonging to *Cyanobacteria*, *Aquificae*, *Nitrospirae*, and *Chloroflexi* formed monophyletic clade, respectively, while those affiliated with *Proteobacteria*, *Firmicutes*, and *Methanobacteria* were distributed across on the phylogenetic tree (Figure 2A, see detailed phylogeny in Supplementary Figure 5).

Jackknifed UPGMA clustering analysis showed that the diazotrophic communities could be divided into the following types: (i) *Nitrospira*-like, accounting for 67-96% of the sequences in Hxq, Jzq, Hs5, Hnt3, and Szt1 hot springs with temperature ranging 54-62°C; (ii) *Aquificae*-like, accounting for 38-85% of the sequences in Srbz2, Hs1, Hxq2, Rhyq2, Rhyq5, Xrd, Srbz3, Hs2, and Hs3 hot springs with temperature ranging 62-84°C; (iii) *Cyanobacteria*-like, accounting for 33-97% of the sequences of Hnt2, Hnt1, Jmq, Srbz1, and Srbz4 hot springs with temperature ranging 34-61°C; (iv) *Nitrospira*- and *Methanobacteria*-like (mainly in Hmz hot spring), belonging to group IV (Figures 2B,C). BLAST analyses revealed that the dominated *Aquificae*-and *Nitrospirae*-like *nifH* gene OTUs were closely related to the genera of *Hydrogenobacter* and *Thermodesulfovibrio*, respectively (Supplementary Table 3). In addition to the above widely existing dominant groups, there were also unique dominant *nifH* gene groups in some of the studied hot springs, such as those affiliated with “*Beta*/*gamma*-*Proteobacteria*” in Hxq1 hot spring, the *Deltaproteobacteria nifH* gene sequences (most of which cannot be assigned to any known genus) in Hnt3, Srbz1 and Hs1 hot springs, and the *Chloroflexi*-like *nifH* gene sequences (associated with the *Roseiflexus* genus) in Hs2 and Jmq hot springs. Furthermore, there were only one or two major OTUs (the relative abundance was above 40%) in most hot springs (Supplementary Figure 6), indicating the main executors of nitrogen fixation process were limited to a few lineages.

### Phylogenetic analysis of *nifH* gene sequences in hot springs of different regions

**FIGURE 3:**
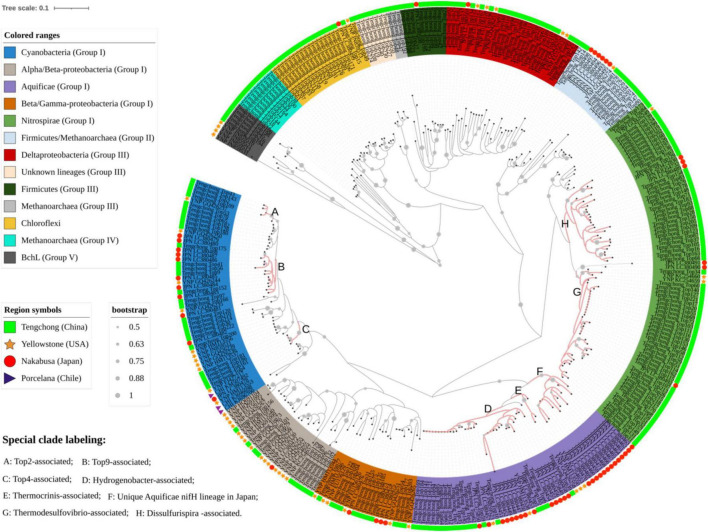
A Neighbor-joining tree showing the phylogenetic relationships between the *nifH* gene sequences obtained in this study and those from Yellowstone, Chile, Japan hot springs.

In order to fully understand the phylogenetic relationships among the *nifH* gene sequences obtained in this study and those from hot springs of Yellowstone, Japan and Chile, phylogenetic analyses were performed by combining the representative *nifH* gene sequences from all of up-to-date known researches of diazotrophs in hot springs (more than 20 hot springs with temperature ranging from 35 to 84°C and pH 2-9; Steunou et al., 2006; Hall et al., 2008; Steunou et al., 2008; Hamilton et al., 2011a,b; Loiacono et al., 2012; Hamilton et al., 2014; Estrella-Alcamán et al., 2015; Alcamán-Arias et al., 2018; Nishihara et al., 2018b,c). The *nifH* gene lineages of hot springs were widely distributed in the phylogenetic tree. Many sequences from the same region were clustered in tight clades within many phylogenetic lineages such as *Cyanobacteria*, *Nitrospirae*, *Proteobacteria* and *Aquificae* (Figure 3). In addition, some clades represented large phylogenetic branches. For example, the group IV and “Unknown lineage” were composed of Yunnan sequences, and some sequences from Japan formed one monophyletic clade of *Aquificae*. This indicates that there are many unique *nifH* gene lineages in the geothermal springs of this region, and the distribution of diazotrophs has biogeographical patterns.

### Influence of environmental factors on the diazotrophic communities

**FIGURE 4:**
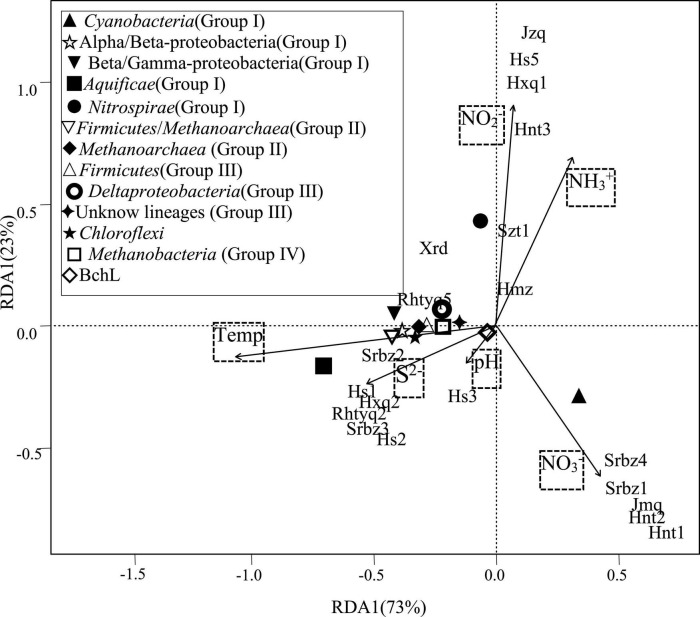
RDA of phylogenetic lineages of *nifH* gene in relation to physicochemical variables. The percent variability explained by each principal component is shown in parentheses in the axis labels.

**FIGURE 5:**
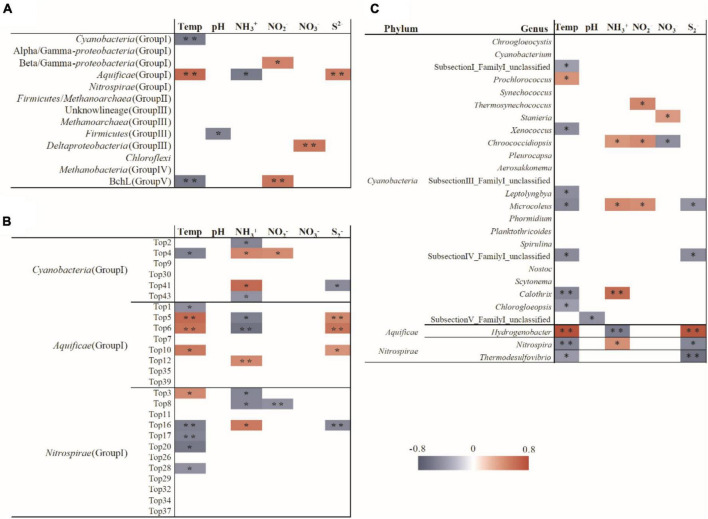
Spearman correlation between the measured environmental variables and the relative abundance of the *nifH* gene lineages **(A)**, *nifH* OTUs **(B)** and the 16S rRNA gene of potential diazotrophic genera **(C)**. ^**^ *p* < 0.01 and * *p* < 0.05.

The redundancy analyses indicated that the diazotrophic communities were influenced by temperature, ammonia, nitrite, nitrate and sulfide in the investigated hot springs. Temperature was significantly correlated with the relative abundance of most phylogenetic lineages of *nifH* gene (Figure 4). Spearman’s correlation analysis showed that the *Aquificae* was positively correlated with temperature and sulfide, but negatively correlated with ammonia. *Cyanobacteria* was negatively correlated with temperature. At the OTU level, many of the *nifH* gene lineages were significantly correlated with temperature, ammonia, and sulfide (Figure 5).

## Discussion

### Diazotrophic diversity in the Tengchong hot springs and comparative analysis with hot springs in other regions

The results of this study proved that active diazotrophs are widely distributed in hot springs, and many diazotrophic lineages have potential nitrogen fixation activity. Although these diazotrophic lineages have been detected by DNA-based methods, they have not been verified by *in situ* expression in hot springs. Furthermore, most of the investigated diazotrophic communities were dominated by the lineages of *Aquificae* and *Nitrospirae*. However, to our best knowledge, the dominance of these lineages has not been reported as the main diazotrophs in other ecosystems. Therefore, it can be referred that hot springs harbored unique active diazotrophs in comparison with other type of ecosystems. Furthermore, the phylogenetic analysis showed remarkable different diazotrophic genetic lineages between hot springs of Tengchong and other regions.

The *nifH* gene of *Aquificae* are mainly derived from *Hydrogenobacter* and *Thermocrinis* (Hamilton et al., 2011a,^2014^; Loiacono et al., 2012), which usually occupy similar niches in high temperature, circumneutral to alkaline terrestrial ecosystems (Eder and Huber, 2002; Reysenbach et al., 2005). YNP hot springs were inhabited by these two genera, but Tengchong hot springs only harbored *Hydrogenobacter* (Supplementary Table 3 and Supplementary Figure 3). The results about 16S rRNA gene in this and previous studies (Song et al., 2012; Hou et al., 2013) also showed the absence of *Thermocrinis* species in Tengchong springs, suggesting the distribution of this diazotrophic genus might have geographical patterns. Interestingly, a large number of 16S rRNA gene sequences belonging to the genus *Thermocrinis* were found in Nakabusa hot springs, Japan, but phylogenetic analysis showed that any *nifH* gene sequences in those hot springs cannot be assigned to *Thermocrinis* (Figure 3; Nishihara et al., 2018b). This suggests that *Thermocrinis*-affiliated bacteria may not have nitrogen fixation ability in some hot springs. This phenomenon can be explained by the fact that some *Thermocrinis* species living in YNP hot springs may obtain *nifH* gene from coexisting diazotrophs such as *Hydrogenobacter* through horizontal gene transfer that was speculated to occur among diazotrophs (Koirala and Brözel, 2021) or prokaryotes in hot springs (Zhu et al., 2019; Li et al., 2021; Qi et al., 2021). In addition, there was one unique *Aquificae*-related *nifH* gene lineage in Japan hot springs (Figure 3), but those related *nifH* gene sequences have stop codons in their amino acid sequences (Nishihara et al., 2018b), indicating they are pseudogenes. So it is reasonable that *Thermocrinis* did not exist or were not expressed in Tengchong hot springs.

The composition of *Nitrospirae* diazotrophs differed between Tengchong and YNP hot springs. *Nitrospirae* diazotrophs were mainly composed of *Thermodesulfovibrio* genus in YNP hot springs (Loiacono et al., 2012; Hamilton et al., 2014; Thiel et al., 2017). In contrast, besides *Thermodesulfovibrio* genus, another *Nitrospirae* diazotrophic lineage belonging to novel genus *Dissulfurispira* was present in Tengchong hot springs (Figure 3). *Dissulfurispira* were recently isolated from Nakabusa hot springs of Japan (Umezawa et al., 2021), and have complete nitrogenase genes (*nifBDEHKX*, genome accession: NZ_AP022873). However, *Dissulfurispira* was not detected in the YNP hot springs. In addition, it is worth noting that the *nifH* gene transcripts in five Tengchong hot springs (Hxq, Jzq, Hs5, Hnt3 and Szt) are dominated by *Nitrospira*, indicating that *Nitrospira* are major active diazotrophs in Tengchong hot springs. However, this phenomenon has never been reported in other regions (Loiacono et al., 2012; Hamilton et al., 2014; Alcamán-Arias et al., 2018). Such difference may be caused by the different community features of the studied hot springs between Tengchong and other regions (Hou et al., 2013). Tengchong hot springs were predominated by *Nitrospira nifH* gene, representing low-temperature and non-phototrophic type of communities (the temperature ranged from 54 to 62 °C; 16S rRNA gene analysis showed that the abundance of phototrophic taxa was below 6%, Supplementary Figure 3). In comparison, Yellowstone and Chile hot springs that have been studied represent either high-temperature chemotrophic or low-temperature phototrophic communities (Loiacono et al., 2012; Hamilton et al., 2014; Alcamán-Arias et al., 2018), in which the *Nitrospira* diazotrophs have weaker adaptability than those coexisting lineages, such as *Aquificae* or *Cyanobacteria*.

A certain amount of *nifH* gene sequences were detected to be related to *Roseiflexus*, a member of *Chloroflexi*, in some samples. Combined with the results of *in situ* studies on YNP hot springs (Loiacono et al., 2012; Klatt et al., 2013), the expression of *nifH* gene of *Roseiflexus* seems to be widespread in hot springs. However, previous studies have pointed out that the lack of *nifEN* gene encoding nitrogenase cofactor assembly protein in *Roseiflexus* (Dos Santos et al., 2012) leads to the unclear understanding of whether the *nifH* gene of *Roseiflexus* spp. encodes functional nitrogenase components. It is believed that “genome downsizing” is one of the important strategies for thermophiles in hot springs to adapt to high temperature (Thomas et al., 2019; Alcorta et al., 2020). So Klatt et al. speculated that *Roseiflexus* species living in hot springs may directly assemble cofactors on the *NifD*/*NifK* heterodimers to offset the loss of *nifEN* gene function (Klatt et al., 2013). Moreover, nitrogenase activity has been demonstrated in organisms lacking *nifEN* gene in *Elusimicrobia* (Zheng et al., 2016). Therefore, the *nifH* gene expression of *Roseiflexus* indicates that the species of this genus has nitrogen fixation activity in hot springs.

### Environmental factors influencing diazotrophs in the tengchong hot springs

In this study, *nifH* gene expression was obtained in Tengchong geothermal springs with a wide range of physicochemical parameters. Combined with the *in situ* expression results of YNP (Steunou et al., 2006; Steunou et al., 2008; Loiacono et al., 2012; Hamilton et al., 2014), Chile (Estrella-Alcamán et al., 2015) and Japanese (Nishihara et al., 2018a) hot springs, it can be seen that the occurrence of biological nitrogen fixation process may not be limited by temperature, pH or geographic location of hot springs. However, some environmental factors may influence the structure of diazotrophic communities in Tengchong hot springs.

Two previous studies demonstrated that temperature influenced the composition of diazotrophs at different locations with the same chemical background in hot spring outflowing channels (Loiacono et al., 2012; Feng et al., 2018). However, no investigation has been performed to assess the contribution of temperature to the shaping of diazotrophic communities among separated hot springs, which generally have different chemistry background. In the investigated samples, the abundance of diazotrophic clades changed regularly with temperature. For example, the *Aquificae*-related *nifH* gene transcripts predominated in the hot springs with relatively high temperature (above 62°C), while *Nitrospira* or *Cyanobacteria* dominated in the hot springs with temperature below 62°C (Figure 2). BLAST analysis showed that the dominant cyanobacterial *nifH* gene OTU “Top2” in high temperature samples (hot springs Hnt1 and Hnt2 with 53-56°C; Figure 2) was most closely related (96% identity) to those retrieved from YNP geothermal springs, while OTU “Top9” was predominant in low-temperature samples (the spring Srbz9 with 34°C), which was similar to the sequences from normal temperature lake environments (96% identity). In addition, the RDA (Figure 4) and Spearman’s correlations analysis (Figure 5) also revealed significant statistical correlation between temperature and composition of diazotrophs. Taken together, temperature represents a strong selective pressure driving the distribution of diazotrophic clades in Tengchong hot springs.

In addition, statistical analyses revealed the abundance of many *nifH* gene OTUs was related to the concentration of ammonia (Figure 5), suggesting the influence of available nitrogen sources on nitrogen-fixating microorganisms (Dixon and Kahn, 2004; Hong et al., 2017; Zilius et al., 2020). Especially, the *Aquificae* diazotrophs showed negative correlation with ammonia, indicating that the expression of *Aquificae*-related *nifH* gene increased with the decrease of ammonia concentration. Furthermore, as far as we known, S^2–^is rarely reported as a factor affecting the active diazotrophic clade in normal environments. However, in the present study, many diazotrophic lineages, especially the *Aquificae*, at OTU and phylum levels showed significant correlations with the concentration of S^2–^(Figure 5), indicating that S^2–^ may affect the distribution of diazotrophs. Such correlations can be explained by the fact that sulfur compounds are common and abundant in hot springs, and they are important electron donors or acceptors and are utilized by many chemolithotrophic organisms to obtain energy (Amenabar and Boyd, 2019). For example, the *Aquificae* strains isolated from Tengchong hot springs can use S^2–^, S^0^, S_2_O_3_^2–^ as the electron donors (Hedlund et al., 2015), so it is reasonable to find positive correlation between S^2–^concentration and *Aquificae nifH* gene abundance (Figure 5). Alternatively, considering that S^2–^ can be easily converted to S^0^ or S_2_O_3_^2–^ by biotical and/or abiotic catalysis in hot springs (Amenabar and Boyd, 2019), the positive correlation between S^2–^concentration and *Aquificae nifH* gene abundance suggested that the oxidation of S^0^ or/and S_2_O_3_^2–^ may be caused by *Aquificae* diazotrophs in the investigated hot springs. At present, it has been proved that the diazotrophic *Aquificae* strains isolated from Nakabusa hot spring in Japan can use S_2_O_3_^2–^ as an electron donor (Nishihara et al., 2018c).

## Conclusion

Biological nitrogen fixation process widely exists in terrestrial geothermal springs. The *nifH* gene transcripts are related to many diazotrophic lineages. The major active diazotrophic lineages belong to *Aquificae*, *Cyanobacteria* and *Nitrospirae*. *Aquificae*- and *Nitrospirae*-like diazotrophs dominate in high- and low-temperature hot springs, respectively. In addition, some investigated springs harbored unique predominant clades. The diazotrophs in Tenchong hot springs exhibited significant difference in phylogenetic lineages from those of YNP and Japanese hot springs. Furthermore, temperature and concentrations of ammonia and sulfide may be important environmental factors influencing the expression and distribution of *nifH* genein hot springs. These results improve the understanding of the distribution and diversity of diazotrophs in hot spring environments.

## Data availability statement

The datasets presented in this study can be found in online repositories. The names of the repository/repositories and accession number(s) can be found in the article/Supplementary material.

## Author contributions

Z-QS, W-JL, and HJ conceived the study. Z-QS, LW, and FL performed the on-site measurements and collected the samples. Z-QS, QZ, and DP analyzed the geochemistry and microbiology of the samples. Z-QS analyzed the sequencing data. Z-QS, W-JL, and HJ drafted the manuscript. All authors reviewed results and commented on the manuscript.

## Footnotes

1
http://secondgenome.com/solutions/resources/data-analysis-tools/piphillin/

2
http://fungene.cme.msu.edu/FunGenePipeline/framebot/form.spr

3
https://www.biosino.org/node/

## Supplementary material

Supplementary Figure 1 Field photos of Tengchong hot springs.

Supplementary Figure 2 Principal component analysis of the measured physicochemical variables of the studied hot springs.

Supplementary Figure 3 Relative abundance of prokaryotic 16S rRNA gene sequences at Phyla levels. Relative abundances of <1% were grouped as “Other.”

Supplementary Figure 4 The abundance of the 16S rRNA gene of potential diazotrophic genera or equal-level unclassified lineages.

Supplementary Figure 5 Neighbor-joining tree showing the detailed phylogenetic affiliation of the OTUs based on protein sequences. Scale bars indicate the Jukes-Cantor distances. Bootstrap values of 50% (for 1,000 iterations) are shown.

Supplementary Figure 6 The abundance of the most 50 abundant *nifH* gene OTUs.

Supplementary Table 1 Description of hot spring samples investigated in this study.

Supplementary Table 2 The NSTI scores of investigated samples.

Supplementary Table 3 The closest GenBank match of the most 50 abundant *nifH* gene OTUs.

## References

[B1] Alcamán-Arias M. E. Pedrós-Alió C. Tamames J. Fernández C. Pérez-Pantoja D. Vásquez M. (2018). Diurnal changes in active carbon and nitrogen pathways along the temperature gradient in Porcelana hot spring microbial mat. *Front. Microbiol.* 9:2353. 10.3389/fmicb.2018.02353 30333812 PMC6176055

[B2] Alcorta J. Alarcón-Schumacher T. Salgado O. Díez B. (2020). Taxonomic novelty and distinctive genomic features of hot spring Cyanobacteria. *Front. Genet.* 11:568223. 10.3389/fgene.2020.568223 33250920 PMC7674949

[B3] Amenabar M. J. Boyd E. S. (2019). A review of the mechanisms of mineral-based metabolism in early Earth analog rock-hosted hydrothermal ecosystems. *World J. Microbiol. Biotechnol.* 35:29. 10.1007/s11274-019-2604-2 30689069

[B4] Bae H. S. Morrison E. Chanton J. P. Ogram A. (2018). Methanogens are major contributors to nitrogen fixation in soils of the *Florida everglades*. *Appl. Environ. Microbiol.* 84:e02222. 10.1128/AEM.02222-17 29374038 PMC5861825

[B5] Bolyen E. Rideout J. R. Dillon M. R. Bokulich N. A. Abnet C. C. Al-Ghalith G. A. (2019). Reproducible, interactive, scalable and extensible microbiome data science using QIIME 2. *Nat. Methods* 37 852–857. 10.1038/s41587-019-0209-9 31341288 PMC7015180

[B6] Calderoli P. A. Collavino M. M. Behrends Kraemer F. Morrás H. J. M. Aguilar O. M. (2017). Analysis of nifH-RNA reveals phylotypes related to Geobacter and *Cyanobacteria* as important functional components of the N2-fixing community depending on depth and agricultural use of soil. *Microbiol. Open* 6:e00502. 10.1002/mbo3.502 28766873 PMC5635172

[B7] Caporaso J. G. Lauber C. L. Walters W. A. Berg-Lyons D. Lozupone C. A. Turnbaugh P. J. (2011). Global patterns of 16S rRNA diversity at a depth of millions of sequences per sample. *Proc. Natl. Acad. Sci. U.S.A.* 108, 4516–4522. 10.1073/pnas.1000080107 20534432 PMC3063599

[B8] Cousins C. R. Fogel M. Bowden R. Crawford I. Boyce A. Cockell C. (2018). Biogeochemical probing of microbial communities in a basalt-hosted hot spring at *Kverkfjöll volcano*, Iceland. *Geobiology* 16 507–521. 10.1111/gbi.12291 29856116

[B9] Dixon R. Kahn D. (2004). Genetic regulation of biological nitrogen fixation. *Nat. Rev. Microbiol.* 2 621–631. 10.1038/nrmicro954 15263897

[B10] Dos Santos P. C. Fang Z. Mason S. W. Setubal J. C. Dixon R. (2012). Distribution of nitrogen fixation and nitrogenase-like sequences amongst microbial genomes. *BMC Genom.* 13:162. 10.1186/1471-2164-13-162 22554235 PMC3464626

[B11] Du J. Liu C. Fu B. Ninomiya Y. Zhang Y. Wang C. (2005). Variations of geothermometry and chemical-isotopic compositions of hot spring fluids in the Rehai geothermal field, southwestern China. *J. Volcanol. Geoth. Res.* 142 243–261. 10.1016/J.JVOLGEORES.2004.11.009

[B12] Eder W. Huber R. (2002). New isolates and physiological properties of the Aquificales and description of *Thermocrinis albus* sp. nov. *Extremophiles* 6 309–318. 10.1007/s00792-001-0259-y 12215816

[B13] Edgar R. C. Haas B. J. Clemente J. C. Quince C. Knight R. (2011). UCHIME improves sensitivity and speed of chimera detection. *Bioinformatics* 27 2194–2200. 10.1093/bioinformatics/btr381 21700674 PMC3150044

[B14] Estrella-Alcamán M. Fernandez C. Delgado A. Bergman B. Díez B. (2015). The cyanobacterium *Mastigocladus fulfills* the nitrogen demand of a terrestrial hot spring microbial mat. *ISME J.* 9 2290–2303. 10.1038/ismej.2015.63 26230049 PMC4579480

[B15] Falkowski P. G. (1997). Evolution of the nitrogen cycle and its influence on the biological sequestration of CO2 in the ocean. *Nature* 387 272–275. 10.1038/387272A0

[B16] Fan X. Y. Gao J. F. Pan K. L. Li D. C. Dai H. H. (2017). Temporal dynamics of bacterial communities and predicted nitrogen metabolism genes in a full-scale wastewater treatment plant. *RSC Adv.* 7 56317–56327. 10.1039/C7RA10704H

[B17] Feng C. Yang J. Jiang H. (2018). Diversity and distribution of nitrogen-fixing Bacteria in two geothermal channels in Tengchong geothermal zone, Yunnan Province. *Earth Sci.* 43 10–18. 10.3799/dqkx.2018.911

[B18] Gaby J. C. Buckley D. H. (2012). A comprehensive evaluation of PCR primers to amplify the nifH gene of nitrogenase. *PLoS One* 7:e42149. 10.1371/journal.pone.0042149 22848735 PMC3405036

[B19] Hall J. R. Mitchell K. R. Jackson-Weaver O. Kooser A. S. Cron B. R. Crossey L. J. (2008). Molecular characterization of the diversity and distribution of a thermal spring microbial community by using rRNA and metabolic genes. *Appl. Environ. Microbiol.* 74 4910–4922. 10.1128/AEM.00233-08 18539788 PMC2519350

[B20] Hamilton T. L. Boyd E. S. Peters J. W. (2011a). Environmental constraints underpin the distribution and phylogenetic diversity of nifH in the Yellowstone geothermal complex. *Microb. Ecol.* 61 860–870. 10.1007/s00248-011-9824-9 21365232

[B21] Hamilton T. L. Koonce E. Howells A. Havig J. R. Jewell T. de la Torre J. R. (2014). Competition for ammonia influences the structure of chemotrophic communities in geothermal springs. *Appl. Environ. Microbiol.* 80 653–661. 10.1128/AEM.02577-13 24242238 PMC3911087

[B22] Hamilton T. L. Lange R. K. Boyd E. S. Peters J. W. (2011b). Biological nitrogen fixation in acidic high-temperature geothermal springs in Yellowstone National Park, Wyoming. *Environ. Microbiol.* 13 2204–2215. 10.1111/j.1462-2920.2011.02475.x 21450003

[B23] He F. Hu X. S. (2005). Hubbell’s fundamental biodiversity parameter and the Simpson diversity index. *Ecol. Lett.* 8, 386–390. 10.1111/J.1461-0248.2005.00729.X

[B24] Hedlund B. Reysenbach A. L. Huang L. Ong J. Liu Z. Dodsworth J. (2015). Isolation of diverse members of the Aquificales from geothermal springs in Tengchong, China. *Front. Microbiol.* 6:3389. 10.3389/fmicb.2015.00157 25774153 PMC4343020

[B25] Hong H. Shen R. Zhang F. Wen Z. Chang S. Lin W. (2017). The complex effects of ocean acidification on the prominent N2-fixing cyanobacterium *Trichodesmium*. *Science* 356 527–531. 10.1126/science.aal2981 28450383

[B26] Hou W. Wang S. Dong H. Jiang H. Briggs B. R. Peacock J. P. (2013). A comprehensive census of microbial diversity in hot springs of Tengchong, Yunnan province China using 16S rRNA gene pyrosequencing. *PLoS One* 8:e53350. 10.1371/journal.pone.0053350 23326417 PMC3541193

[B27] Hu J. Richwine J. D. Keyser P. D. Li L. Yao F. Jagadamma S. (2021). Nitrogen fertilization and native C4 grass species alter abundance, activity, and diversity of soil diazotrophic communities. *Front. Microbiol.* 12:675693. 10.3389/fmicb.2021.675693 34305840 PMC8297707

[B28] Iwai S. Weinmaier T. Schmidt B. L. Albertson D. G. Poloso N. J. Dabbagh K. (2016). Piphillin: improved prediction of metagenomic content by direct inference from human microbiomes. *PLoS One* 11:e0166104. 10.1371/journal.pone.0166104 27820856 PMC5098786

[B29] Jiang H. Dong H. Zhang G. Yu B. Chapman L. R. Fields M. W. (2006). Microbial diversity in water and sediment of Lake Chaka: an athalassohaline lake in northwestern China. *Appl. Environ. Microbiol.* 72 3832–3845. 10.1128/AEM.02869-05 16751487 PMC1489620

[B30] Jiao J. Y. Fu L. Hua Z. S. Liu L. Salam N. Liu P. F. (2021). Insight into the function and evolution of the Wood–Ljungdahl pathway in Actinobacteria. *ISME J.* 15 3005–3018. 10.1038/s41396-021-00935-9 33953361 PMC8443620

[B31] Jiao J. Y. Liu L. Hua Z. S. Fang B. Z. Zhou E. M. Salam N. (2020). Microbial dark matter coming to light: challenges and opportunities. *Natl. Sci. Rev.* 8:280. 10.1093/nsr/nwaa280 34691599 PMC8288357

[B32] Klatt C. G. Liu Z. Ludwig M. Kühl M. Jensen S. I. Bryant D. A. (2013). Temporal metatranscriptomic patterning in phototrophic Chloroflexi inhabiting a microbial mat in a geothermal spring. *ISME J.* 7 1775–1789. 23575369 10.1038/ismej.2013.52 PMC3749495

[B33] Koirala A. Brözel V. S. (2021). Phylogeny of nitrogenase structural and assembly components reveals new insights into the origin and distribution of nitrogen fixation across Bacteria and Archaea. *Microorganisms* 9:1662. 10.3390/microorganisms9081662 34442741 PMC8399215

[B34] Langille M. G. I. Zaneveld J. Caporaso J. G. McDonald D. Knights D. Reyes J. A. (2013). Predictive functional profiling of microbial communities using 16S rRNA marker gene sequences. *Nat. Biotechnol.* 31 814–821. 10.1038/nbt.2676 23975157 PMC3819121

[B35] Larson C. A. Mirza B. Rodrigues J. L. M. Passy S. I. (2018). Iron limitation effects on nitrogen-fixing organisms with possible implications for cyanobacterial blooms. *FEMS Microbiol. Ecol.* 94:fiy046. 10.1093/femsec/fiy046 29566225

[B36] Letunic I. Bork P. (2019). Interactive Tree Of Life (iTOL) v4: recent updates and new developments. *Nucleic Acids Res.* 47 W256–W259. 10.1093/nar/gkz239 30931475 PMC6602468

[B37] Li Y. X. Rao Y. Z. Qi Y. L. Qu Y. N. Chen Y. T. Jiao J. Y. (2021). Deciphering symbiotic interactions of “Candidatus Aenigmarchaeota” with inferred horizontal gene transfers and co-occurrence networks. *mSystems* 6:e0060621. 10.1128/mSystems.00606-21 34313464 PMC8407114

[B38] Lin K. H. Liao B. Y. Chang H. W. Huang S. W. Chang T. Y. Yang C. Y. (2015). Metabolic characteristics of dominant microbes and key rare species from an acidic hot spring in Taiwan revealed by metagenomics. *BMC Genomics* 16:1029. 10.1186/s12864-015-2230-9 26630941 PMC4668684

[B39] Loiacono S. T. Meyer-Dombard D. R. Havig J. R. Poret-Peterson A. T. Hartnett H. E. Shock E. L. (2012). Evidence for high-temperature in situ nifH transcription in an alkaline hot spring of Lower Geyser Basin, Yellowstone National Park. *Environ. Microbiol.* 14 1272–1283. 10.1111/j.1462-2920.2012.02710.x 22404902

[B40] Magoč T. Salzberg S. L. (2011). FLASH: fast length adjustment of short reads to improve genome assemblies. *Bioinformatics* 27 2957–2963. 10.1093/bioinformatics/btr507 21903629 PMC3198573

[B41] Meyer M. Stenzel U. Hofreiter M. (2008). Parallel tagged sequencing on the 454 platform. *Nat. Protoc.* 3 267–278. 10.1038/nprot.2007.520 18274529

[B42] Meyer-Dombard D. R. Shock E. L. Amend J. P. (2005). Archaeal and bacterial communities in geochemically diverse hot springs of Yellowstone National Park, USA. *Geobiology* 3 211–227. 10.1111/gbi.12051 23981055

[B43] Miller S. R. Purugganan M. D. Curtis S. E. (2006). Molecular population genetics and phenotypic diversification of two populations of the thermophilic cyanobacterium *Mastigocladus laminosus*. *Appl. Environ. Microbiol.* 72 2793–2800. 10.1128/AEM.72.4.2793-2800.2006 16597984 PMC1449082

[B44] Moisander P. H. Serros T. Paerl R. W. Beinart R. A. Zehr J. P. (2014). Gammaproteobacterial diazotrophs and nifH gene expression in surface waters of the South Pacific Ocean. *ISME J.* 8 1962–1973. 10.1038/ismej.2014.49 24722632 PMC4184014

[B45] Nishihara A. Haruta S. McGlynn S. E. Thiel V. Matsuura K. (2018a). Nitrogen fixation in thermophilic chemosynthetic microbial communities depending on hydrogen, sulfate, and carbon dioxide. *Microbes Environ.* 33 10–18. 10.1264/jsme2.ME17134 29367473 PMC5877335

[B46] Nishihara A. Matsuura K. Tank M. McGlynn S. E. Thiel V. Haruta S. (2018b). Nitrogenase activity in thermophilic chemolithoautotrophic bacteria in the phylum Aquificae isolated under nitrogen-fixing conditions from Nakabusa hot springs. *Microbes Environ.* 33 394–401. 10.1264/jsme2.ME18041 30473565 PMC6307999

[B47] Nishihara A. Thiel V. Matsuura K. McGlynn S. E. Haruta S. (2018c). Phylogenetic diversity of nitrogenase reductase genes and possible nitrogen-fixing bacteria in thermophilic chemosynthetic microbial communities in Nakabusa hot springs. *Microbes Environ.* 3 357–365. 10.1264/jsme2.ME18030 30404970 PMC6307998

[B48] Qi Y. L. Evans P. N. Li Y. X. Rao Y. Z. Qu Y. N. Tan S. (2021). Comparative genomics reveals thermal adaptation and a high metabolic diversity in “*Candidatus bathyarchaeia*.”. *mSystems* 6:e0025221. 10.1128/mSystems.00252-21 34282939 PMC8407382

[B49] Quast C. Pruesse E. Yilmaz P. Gerken J. Schweer T. Yarza P. (2013). The SILVA ribosomal RNA gene database project: improved data processing and web-based tools. *Nucleic Acids Res.* 41 D590–D596. 10.1093/nar/gks1219 23193283 PMC3531112

[B50] Raymond J. Siefert J. L. Staples C. R. Blankenship R. E. (2004). The natural history of nitrogen fixation. *Mol. Biol. Evol.* 21 541–554. 10.1093/molbev/msh047 14694078

[B51] Reysenbach A. L. Banta A. Civello S. Daly J. Mitchell K. Lalonde S. (2005). “The Aquificales in Yellowstone National Park,” in *geothermal biology and geochemistry in Yellowstone National Park*, eds Inskeep W. P. McDermott T. R. (Bozeman, MT: Thermal Biology Institute, Montana State University), 129–142.

[B52] Roehr J. T. Dieterich C. Reinert K. (2017). Flexbar 3.0-SIMD and multicore parallelization. *Bioinformatics* 33 2941–2942. 10.1093/bioinformatics/btx330 28541403

[B53] Schloss P. D. Westcott S. L. Ryabin T. Hall J. R. Hartmann M. Hollister E. B. (2009). Introducing mothur: open-source, platform-independent, community-supported software for describing and comparing microbial communities. *Appl. Environ. Microbiol.* 75 7537–7541. 10.1128/AEM.01541-09 19801464 PMC2786419

[B54] Sogin M. L. Morrison H. G. Huber J. A. Welch D. M. Huse S. M. Neal P. R. (2006). Microbial diversity in the deep sea and the underexplored “rare biosphere.”. *Proc. Natl. Acad. Sci. U.S.A.* 103 12115–12120. 10.1073/pnas.0605127103 16880384 PMC1524930

[B55] Song Z. Q. Wang F. P. Zhi X. Y. Chen J. Q. Zhou E. M. Liang F. (2012). Bacterial and archaeal diversities in Yunnan and Tibetan hot springs, China. *Environ. Microbiol.* 15 1160–1175. 10.1111/1462-2920.12025 23126508

[B56] Spellerberg I. F. Fedor P. J. (2003). A tribute to claude shannon (1916-2001) and a plea for more rigorous use of species richness, species diversity and the ‘Shan-Non-Wiener’ index. *Glob. Ecol. Biogeogr.* 12 177–179. 10.1046/j.1466-822X.2003.00015.x

[B57] Steunou A. S. Bhaya D. Bateson M. M. Melendrez M. C. Ward D. M. Brecht E. (2006). In situ analysis of nitrogen fixation and metabolic switching in unicellular thermophilic cyanobacteria inhabiting hot spring microbial mats. *Proc. Natl. Acad. Sci. U.S.A.* 103 2398–2403. 10.1073/pnas.0507513103 16467157 PMC1413695

[B58] Steunou A. S. Jensen S. I. Brecht E. Becraft E. D. Bateson M. M. Kilian O. (2008). Regulation of nif gene expression and the energetics of N2 fixation over the diel cycle in a hot spring microbial mat. *ISME J.* 2 364–378. 10.1038/ismej.2007.117 18323780

[B59] Thiel V. Hügler M. Ward D. M. Bryant D. A. (2017). The dark side of the mushroom spring microbial mat: life in the shadow of chlorophototrophs. II. metabolic functions of abundant community members predicted from metagenomic analyses. *Front. Microbiol.* 8:943. 10.3389/fmicb.2017.00943 28634470 PMC5459899

[B60] Thomas S. C. Tamadonfar K. O. Seymour C. O. Lai D. Dodsworth J. A. Murugapiran S. K. (2019). Position-specific metabolic probing and metagenomics of microbial communities reveal conserved central carbon metabolic network activities at high temperatures. *Front. Microbiol.* 10:3389. 10.3389/fmicb.2019.01427 31333598 PMC6624737

[B61] Umezawa K. Kojima H. Kato Y. Fukui M. (2021). *Dissulfurispira thermophila* gen. nov., sp. nov., a thermophilic chemolithoautotroph growing by sulfur disproportionation, and proposal of novel taxa in the phylum Nitrospirota to reclassify the genus *Thermodesulfovibrio*. *Syst. Appl. Microbiol.* 44:126184. 10.1016/j.syapm.2021.126184 33676265

[B62] Wang K. Ye X. Zhang H. Chen H. Zhang D. Liu L. (2016). Regional variations in the diversity and predicted metabolic potential of benthic prokaryotes in coastal northern Zhejiang, East China Sea. *Sci. Rep.* 6:38709. 10.1038/srep38709 27917954 PMC5137025

[B63] Xian W. D. Salam N. Li M. M. Zhou E. M. Yin Y. R. Liu Z. T. (2020). Network-directed efficient isolation of previously uncultivated Chloroflexi and related bacteria in hot spring microbial mats. *NPJ Biofilms Microbiomes* 6:20. 10.1038/s41522-020-0131-4 32350263 PMC7190741

[B64] Zehr J. P. Jenkins B. D. Short S. M. Steward G. F. (2003). Nitrogenase gene diversity and microbial community structure: a cross-system comparison. *Environ. Microbiol.* 5 539–554. 10.1046/j.1462-2920.2003.00451.x 12823187

[B65] Zheng H. Dietrich C. Radek R. Brune A. (2016). *Endomicrobium proavitum*, the first isolate of *Endomicrobia* class. nov. (phylum Elusimicrobia) an ultra microbacterium with an unusual cell cycle that fixes nitrogen with a Group IV nitrogenase. *Environ. Microbiol.* 18 191–204. 10.1111/1462-2920.12960 26119974

[B66] Zhu Q. Mai U. Pfeiffer W. Janssen S. Asnicar F. Sanders J. G. (2019). Phylogenomics of 10,575 genomes reveals evolutionary proximity between domains Bacteria and Archaea. *Nat. Commun.* 10:5477. 10.1038/s41467-019-13443-4 31792218 PMC6889312

[B67] Zilius M. Samuiloviene A. Stanislauskienë R. Broman E. Bonaglia S. Meškys R. (2020). Depicting temporal, functional, and phylogenetic patterns in estuarine diazotrophic communities from environmental DNA and RNA. *Microb. Ecol.* 81 36–51. 10.1007/s00248-020-01562-1 32803362

