# Supplementary material for: nifH gene expression and diversity in geothermal springs of Tengchong, China
Source: Front Microbiol. 2022 Sep 8;13:980924. doi: 10.3389/fmicb.2022.980924 (PMC9493357; doi:10.3389/fmicb.2022.980924)
Supplement: Supplementary Figure 1 — Field photos of Tengchong hot springs. [file Presentation_1.pdf]

Supplementary Table S1 Description of hot spring samples investigated in this study.

Sample name	Sample code	GPS Locations	Descriptions
Hamazui	Hmz	E 98.445483<br>N 24.953036	A pool with slow outflow. Irregularly shaped and gray mats.
Hashang#1	Hs1	E 98.445438<br>N 24.952925	Small source pool with high flow clastic stone lied on the bottom. Some thin streamer with colors of pink are formed on the sample.
Hashang#2	Hs2	E 98.445447<br>N 24.952933	A shallow flow stream downstream of Hs1. Some thin streamer with colors of gray are formed on the sample.
Hashang#3	Hs3	E 98.445456<br>N 24.952958	A shallow flow stream downstream of Hs2. Some thin streamer with colors of gray are formed on the sample.
Hashang#5	Hs5	E 98.445477<br>N 24.952987	Roughly square pool,~35 cm depth. Clear water, slight degassing sources. Darkblack mud formed on the bottom.
Heinitan#1	Hnt1	E 98.138010<br>N 25.349960	A rectangle pool with ~3m length, ~2.5m wide and ~3m deep. Clear water and high flow. Green and yellow mats formed on the bottom of hot spring.
Heinitan#2	Hnt2	E 98.138072<br>N 25.349041	Roughly square pool with ~90cm length and ~90 cm depth. Green and pink mat formed on the hot spring.
Heinitan#3	Hnt3	E 98.138072<br>N 25.349033	Roughly square pool with ~8m length,~35 cm depth. Clear water, slight degassing sources. Black mud formed on the bottom.
Huanxiquan#1	Hxq1	E 98.348014<br>N 25.178390	A roughly elliptic pool with ~110cm length, ~50cm width and ~65 cm depth. Clear water， gray sandy sediments， and slight degassing sources.
Huanxiquan#2	Hxq2	E 98.348003<br>N 25.179054	A rectangle pool with ~60cm length and ~40cm wide. Covered with a concrete lid, gray and brown sandy sediments.
Jiemingquan	Jmq	E 98.466989<br>N 25.442802	Roughly square pool with ~2m length, ~2.5m depth and covered with a concrete lid. Dark green and black mud sediments.
Jinzequan	Jzq	E 98.467326<br>N 25.443377	A square pool with side ~ 2.5m length and depth 1.5m. Dark green mud formed on the bottom
Xianrendong	Xrd	E 98.466805<br>N 25.441404	A square pool with side ~ 1.0m length, ~ 0.5m wide and 0.7m depth. gray and black sandy sediments.
Rehaitiyanqu#2	Rhtyq2	E 24.571273<br>N 98.261745	Small pool with slow outflow and irregularly shaped. Black water from suspended clays, silicate sands at bottom.
Rehaitiyanqu#5	Rhtyq5	E 24.571271<br>N 98.261744	Small acid pool with slow outflow, circular shaped. Black water from suspended clays. Also silicate sands at bottom.
Shizitou#1	Szt1	E 98.445357<br>N 24.953085	Irregularly shaped pool with many bubbling sources. Clear water and gray mud sediments.
Shuirebaozha#1	Srbz1	N 24.950001<br>E 98.437211	Small source pool with many bubbling sources. Clear water and gray mud sediments. Some thin biofilms with colors of light green are formed on the sample.
Shuirebaozha#2	Srbz2	E 98.437212<br>N 24.949991	A source pool with high flow, clastic stone lied on the bottom, some thin biofilms with colors of black and gray are formed on the sample.
Shuirebaozha#3	Srbz3	E 98.437219<br>N 24.949985	A source pool with high flow and clastic stone lied on the bottom. Some thin biofilms with colors of gray are formed on the sample.
Shuirebaozha#4	Srbz4	N 24.950009<br>E 98.437202	A small source pool with many bubbling sources. Clear water and green and brown mat.

Supplementary Table S2 The NSTI scores of investigated samples

Sample code	NSTI score
Hs1	0.06
Srbz2	0.06
Srbz3	0.11
Hmz	0.28
Hs2	0.13
Hxq2	0.16
Rhtyq5	0.15
Xrd	0.28
Hs3	0.16
Rhtyq2	0.08
Szt1	0.16
Jzq	0.27
Srbz1	0.17
Hnt2	0.05
Jmq	0.15
Hnt3	0.16
Hnt1	0.17
Hxq1	0.20
Hs5	0.16
Srbz4	0.13

Supplementary Table S3. The closest GenBank match of the most 50 abundant *nifH* gene OTUs.

**OTU ID**	**Closest GenBank match (accession number)**	**% Similarity**	**Lineages**
Top2	*Cronbergiasiamensis*NQAIF308(AJF93430)	91	*Cyanobacteria*(Group I)
Top4	Richeliacyanobiont of Rhizosolenia (AEB97743)	92	
Top9	Cyanobacteriumscsio T-2(ALC78854)	96	
Top30	*Oscillatoria*sp.(AAX82506)	95	
Top41	Cyanobacterium scsio T-2(ALC78854)	99	
Top43	*Cronbergiasiamensis*NQAIF308(AJF93430)	90	
Top31	*Agrobacterium tumefaciens*(ACN88695)	99	Beta/Gamma-proteobacteria(Group I)
Top1	*Hydrogenobacterthermophilus*(WP_012963773)	94	*Aquificae*(Group I)
Top5	*Hydrogenobacter* sp. (BBD74536)	95	
Top6	*Hydrogenobacter* sp. (BBD74535)	92	
Top7	*Hydrogenobacter* sp. (BBD74536)	97	
Top10	*Hydrogenobacter* sp. (BBD74536)	97	
Top12	*Hydrogenobacter* sp. (BBD74536)	98	
Top35	*Hydrogenobacterthermophilus*(WP_012963773)	94	
Top39	*Hydrogenobacterthermophilus*(WP_012963773)	100	
Top3	*Nitrospirae* bacterium (NOY64614)	93	*Nitrospirae*(Group I)
Top8	*Thermodesulfovibrioaggregans* (WP_059176981)	100	
Top11	*Thermodesulfovibrio* sp. (WP_012546613)	93	
Top16	*Nitrospirae* bacterium (OGW45220)	93	
Top17	*Thermodesulfovibrio* sp. (WP_012546613)	94	
Top20	*Thermodesulfovibrio* sp. (WP_012546613)	95	
Top26	*Thermodesulfovibrio* sp. (WP_012546613)	90	
Top28	*Thermodesulfovibrio* sp. (WP_012546613)	90	
Top29	*Nitrospirae* bacterium (RJQ54732)	90	
Top32	*Thermodesulfovibrio* sp. (WP_012546613)	93	
Top34	*Thermodesulfovibrio* sp. (WP_012546613)	100	
Top37	*Thermodesulfovibrioaggregans* (WP_059176981)	98	
Top14	*Caldicellulosiruptormorganii*(WP_045170428)	98	*Firmicutes*/*Methanoarchaea*(Group II)
Top19	Unidentified bacterium(AAC36027)	78	Unknow lineage(Group III)
Top40	*Acetobacteriumbakii*(WP_050740289)	77	
Top50	Unidentified bacterium(AAC36027)	78	
Top48	Unidentified bacrerium(BAA28452)	93	*Methanoarchaea*(Group III)
Top21	*Sporomusaceae*sp.(WP_018701495)	93	*Firmicutes*(Group III)
Top27	*Selenomonasruminantium*(WP_073090165)	90	
Top42	*Sporomusaceae*sp.(WP_018701495)	93	
Top47	*Clostridiumclariflavum* (WP_027622762)	96	
Top24	*Desulfomicrobiumbaculatum*(AAL06265)	90	*Deltaproteobacteria*(Group III)
Top25	*Treponemazuelzerae*(AAK01223)	88	
Top15	*Roseiflexuscastenholzii*(WP_012122497)	99	*Chloroflexi*
Top23	*Roseiflexuscastenholzii*(WP_012122497)	93	
Top33	*Roseiflexus*sp.(WP_011955961)	95	
Top46	*Roseiflexuscastenholzii*(WP_012122497)	81	
Top49	*Roseiflexuscastenholzii*(WP_012122497)	96	
Top13	*Methanothermobactermarburgensi*s(WP_013295840)	96	*Methanobacteria* (Group IV)
Top18	Methanothermobactermarburgensis(WP_013295840)	95	
Top22	Methanothermobactermarburgensis(WP_013295840)	96	
Top36	*Methanobacteriumpaludism*(WP_013825083)	80	
Top38	*Methanobacteriumpaludism*(WP_013825083)	79	
Top44	*Methanosphaeracuniculi*(WP_095608939)	70	
Top45	*Bosea*sp.(OYW63368)	94	BchL(GroupV)

Supplementary Figure captions

Supplementary Figure 1. Field photos of Tengchong hot springs.

Supplementary Figure 2. Principal component analysis of the measured physicochemical variables of the studied hot springs.

Supplementary Figure 3: Relative abundance of prokaryotic 16S rRNA gene sequences at Phyla levels. Relative abundances of <1% were grouped as “Other.”

Supplementary Figure 4. The abundance of the 16S rRNA gene of potential diazotrophic genera or equal-level unclassified lineages.

Supplementary Figure 5. Neighbor-joining tree showing the detailed phylogenetic affiliation of the OTUs based on protein sequences. Scale bars indicate the Jukes-Cantor distances. Bootstrap values of 50% (for 1000 iterations) are shown.

Supplementary Figure 6. The abundance of the most 50 abundant *nifH* gene OTUs.

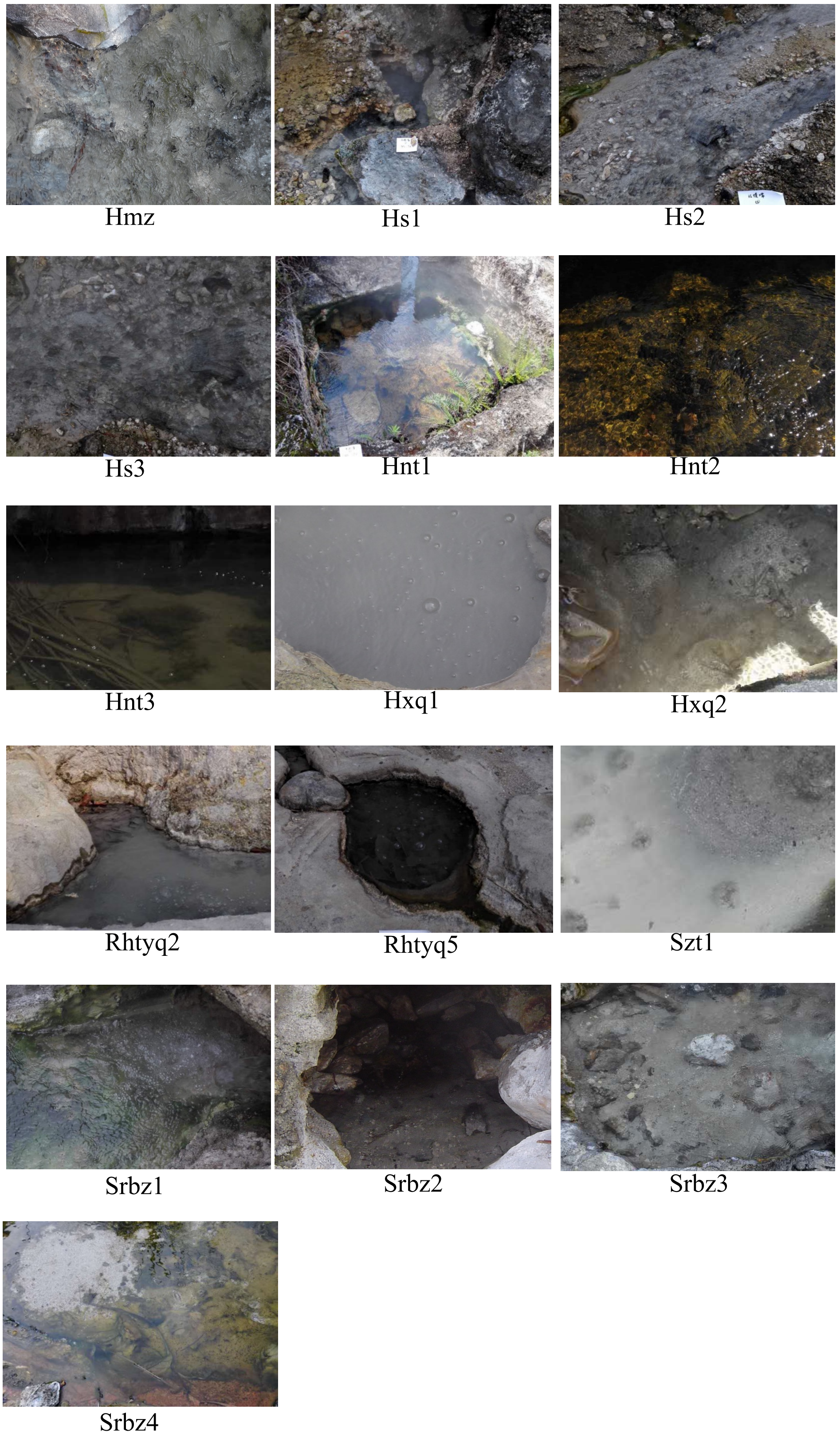

Supplementary Figure 1. Field photos of Tengchong hot springs.

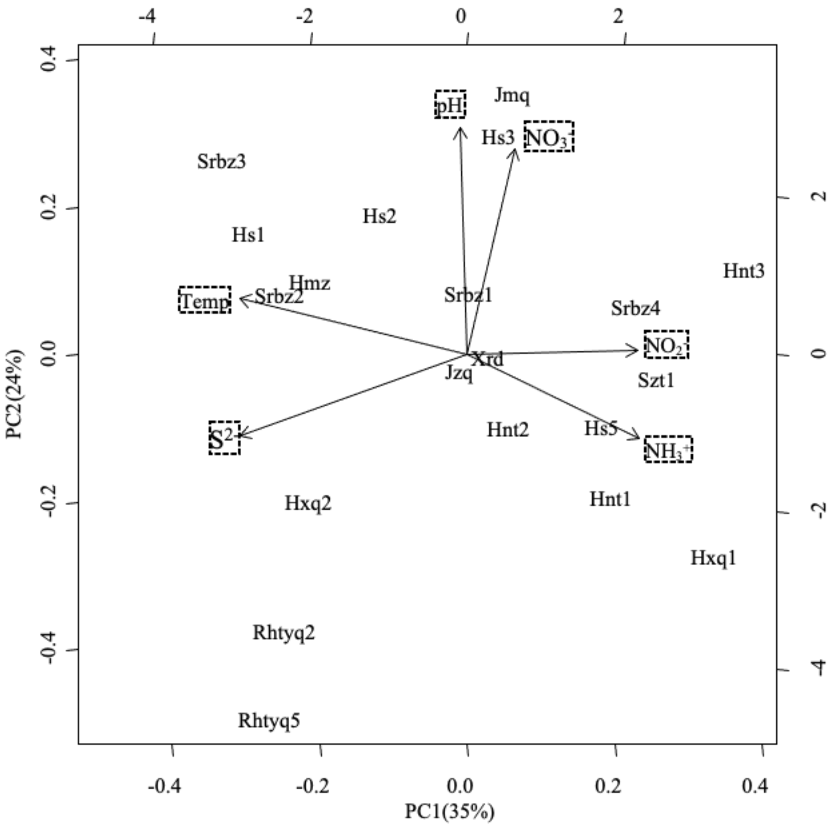

Supplementary Figure 2. Principal component analysis of the measured physicochemical variables of the studied hot springs.

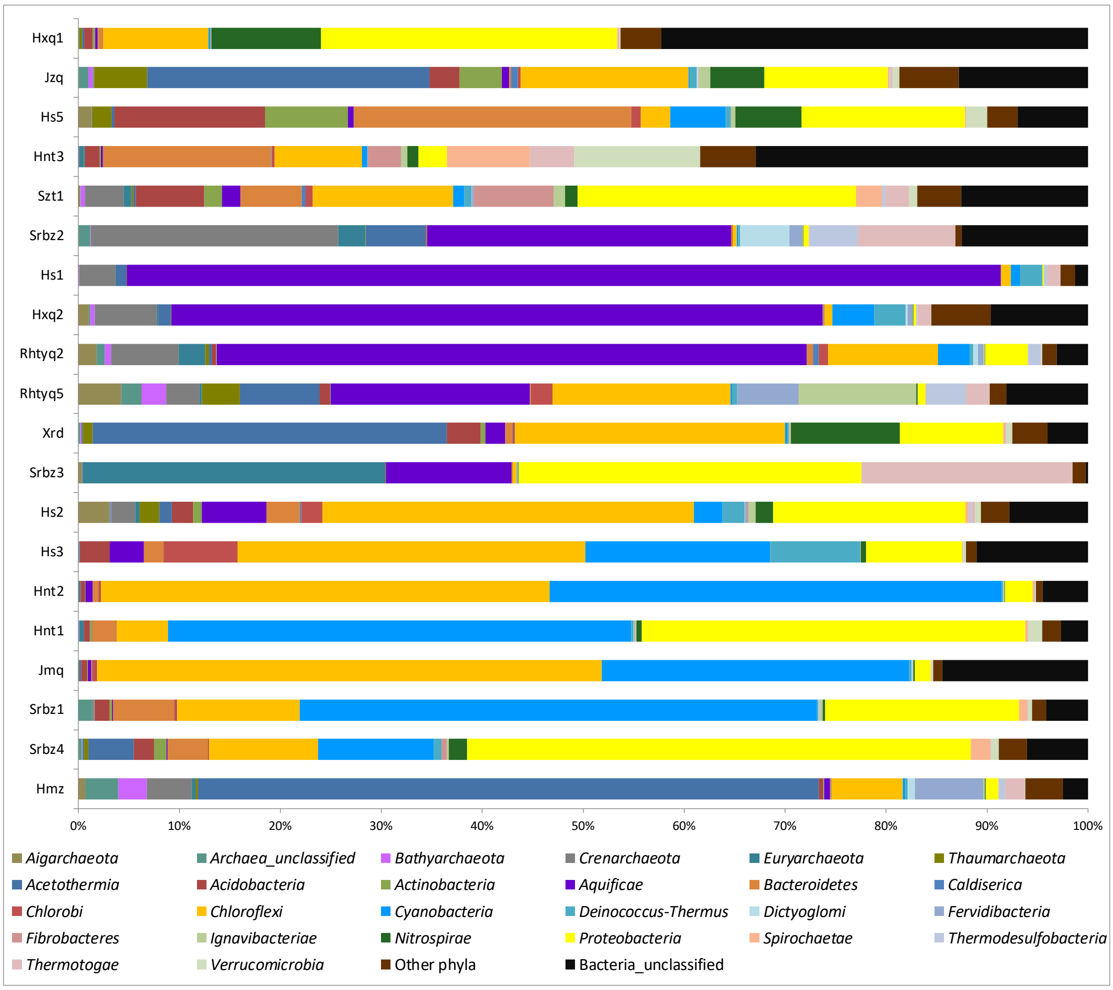

Supplementary Figure 3. Relative abundance of prokaryotic 16S rRNA gene sequences at Phyla levels. Relative abundances of <1% were grouped as "Other."

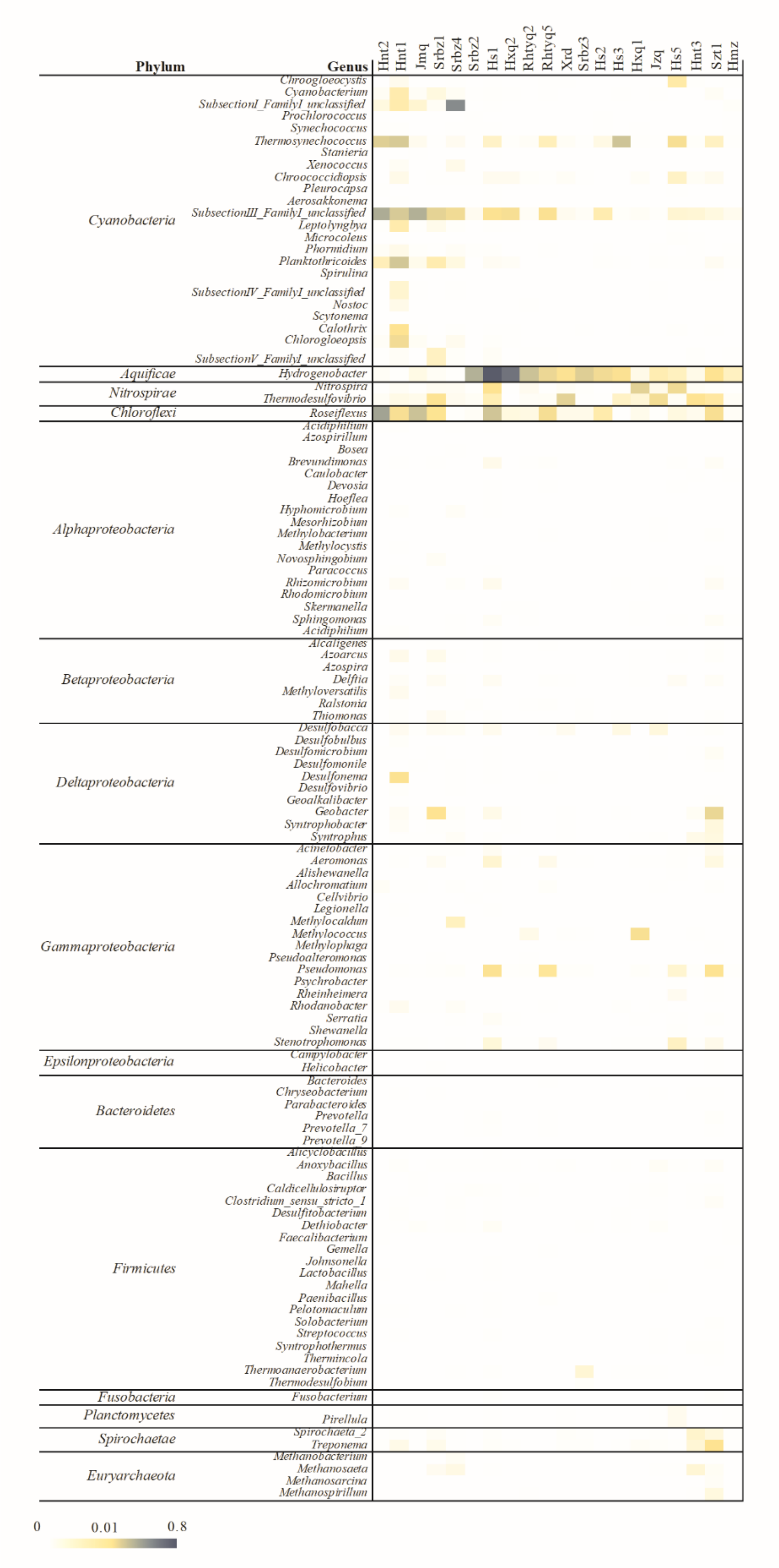

Supplementary Figure 4. The abundance of the 16S rRNA gene of potential diazotrophic genera or equal-level unclassified lineages.

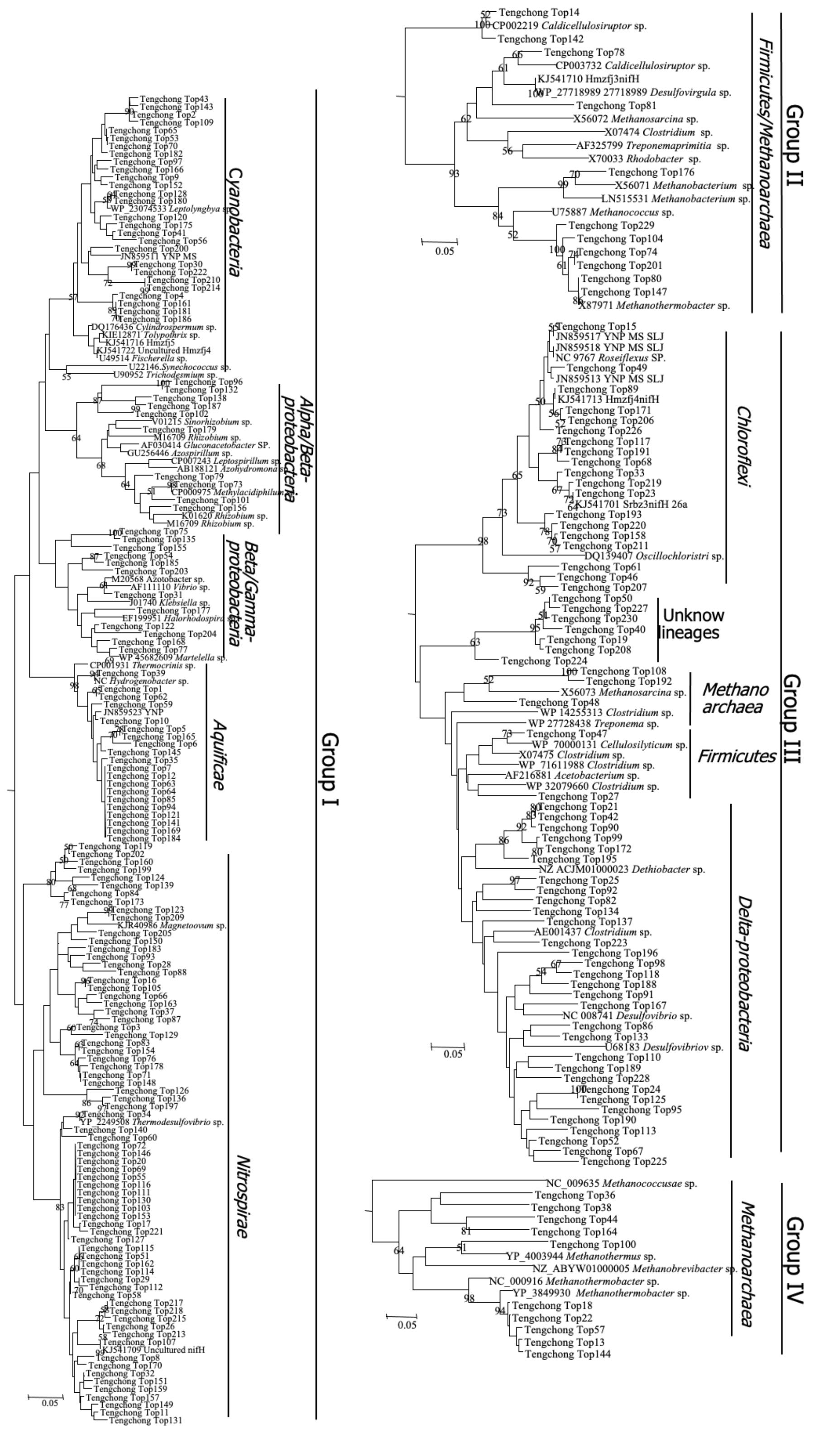

Supplementary Figure 5. Neighbor-joining tree showing the detailed phylogenetic affiliation of the OTUs based on protein sequences. Scale bars indicate the Jukes-Cantor distances. Bootstrap values of 50% (for 1000 iterations) are shown.

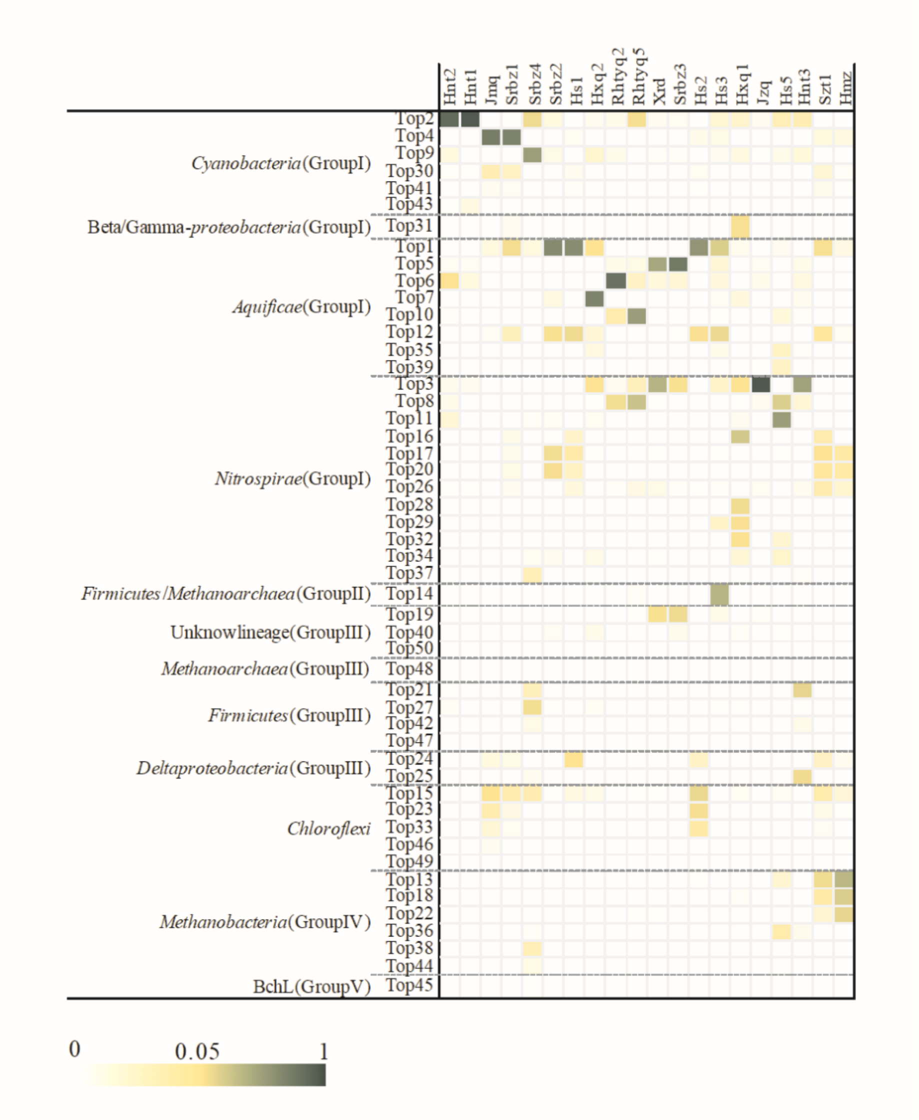

Supplementary Figure 6. The abundance of the most 50 abundant *nifH* gene OTUs.
